# The Use of IL-1 Receptor Antagonist (Anakinra) in Idiopathic Recurrent Pericarditis: A Narrative Review

**DOI:** 10.1155/2016/7840724

**Published:** 2016-01-31

**Authors:** Shankar Baskar, Allan L. Klein, Andrew Zeft

**Affiliations:** ^1^The Heart Institute, Cincinnati Children's Hospital Medical Center, 3333 Burnet Avenue, Cincinnati, OH 45229, USA; ^2^Heart & Vascular Institute, Department of Cardiovascular Imaging, Cleveland Clinic, 9500 Euclid Avenue, Cleveland, OH 44195, USA; ^3^Section of Pediatric Rheumatology, Cleveland Clinic, 9500 Euclid Avenue, Cleveland, OH 44195, USA

## Abstract

Recurrent pericarditis is a complication of acute pericarditis in 20–30% of the patients and is usually idiopathic in nature. The underlying pathogenesis of this condition remains unclear, although immune-mediated mechanisms seem likely. A subgroup of these patients with refractory symptoms can be challenging to manage, and multiple immunosuppressive medications have been used without consistent benefit. Anakinra, an interleukin-1 receptor antagonist, has been used in treatment of rheumatoid arthritis and autoinflammatory syndromes. Preliminary evidence suggests that anakinra could be a promising therapy for idiopathic recurrent pericarditis. In this narrative review, we summarize the current understanding of the etiopathogenesis of idiopathic recurrent pericarditis, mechanism of action of anakinra, and the preliminary evidence, supporting the use of anakinra in pericarditis.

## 1. Introduction

Recurrent pericarditis is a common complication of acute pericarditis and affects 20–30% of patients after an initial attack [1]. It is characterized by the recurrence of signs and symptoms of pericarditis after a symptom-free interval of at least 6 weeks. Diagnosis is based on the presence of typical chest pain (sharp and pleuritic in nature, improved by sitting up and leaning forward) along with 1 or more of the following signs: fever, pericardial friction rub, electrocardiographic changes, echocardiographic evidence of pericardial effusion, and elevated markers of inflammation (white blood cell count, C-reactive protein, or erythrocyte sedimentation rate) [1]. Colchicine remains the mainstay therapy, but a subset of patients have refractory symptoms or are steroid dependent. Anakinra, an interleukin-1 receptor antagonist, has been used in the treatment of rheumatoid arthritis and autoinflammatory syndromes and could be a promising therapy for idiopathic recurrent pericarditis (IRP). In this review, we will discuss the etiopathogenesis of recurrent pericarditis, anakinra's mechanism of action, and preliminary studies supporting its use in the treatment of IRP.

## 2. Etiopathogenesis of Recurrent Pericarditis

The underlying etiology of recurrent pericarditis is poorly understood. In a subset of cases, a viral mediated pathogenesis may be determined. However, no specific etiology is found in most patients, leading to a diagnosis of IRP [1]. Multiple hypotheses have been proposed to explain the pathogenesis of IRP. Increased recurrences of pericarditis episodes in patients with IRP with the use of corticosteroids are suggestive of an unidentified viral infection due to the increased viral replication associated with steroid therapy. However, misdirected innate and adaptive immune responses are believed to play a key role in the pathogenesis of IRP [2]. A growing body of evidence suggests that these abnormal immune responses consist of both autoimmune and autoinflammatory pathogenic processes [3, 4].

The activation of the adaptive immune system via the innate immune system and the loss of tolerance characterize autoimmune diseases, while innate immune system is the major effector in autoinflammatory diseases [4]. The pathologic role of autoimmune processes in IRP is supported by recurrent pericarditis that occurs in autoimmune conditions such as systemic lupus erythematous, presence of heart-specific antibodies, increased prevalence of anti-nuclear antibodies, and human leukocyte antigen (HLA) haplotype specificity in patients with IRP [3, 5–7]. Self-antigens that are exposed after an acute pericarditis attack along with toll-like receptor activation are thought to act as targets for adaptive immune responses eliciting an autoimmune reaction [3].

Patients with autoinflammatory diseases such as familial Mediterranean fever (FMF) and tumor necrosis factor receptor associated periodic syndrome (TRAPS) have mutations in inflammasome-related proteins (a subset of intracellular pattern recognition receptors), which results in an abnormal innate immune response, leading to bouts of recurrent pericarditis [3]. In fact, pericarditis is considered to be the most frequent cardiac manifestation of both FMF and TRAPS [8]. Low penetrance variants of genes coding for the tumor necrosis factor superfamily have also been associated with recurrent pericarditis [9]. The presence of proinflammatory cytokines in the pericardial fluid of IRP patients lends direct support to both an autoimmune and/or autoinflammatory etiopathogenesis [10].

## 3. Treatment of Idiopathic Recurrent Pericarditis

Treatment strategies have evolved based on our understanding of these immunopathogenic hypotheses. Traditionally, steroids have been used based on our presumed knowledge of the autoimmune pathology. Colchicine is also used based on its studied efficiency in treating patients with FMF [11, 12]. Clinicians increasingly use colchicine in an effort to prevent the use of high dose corticosteroids in IRP patients. However, 5% of IRP patients require high or prolonged courses of corticosteroids and experience medication side effects. This group of refractory IRP patients are challenging to manage and are often treated with disease-modifying antirheumatic drugs (DMARDs) such as methotrexate or immunomodulators such as azathioprine for their presumed efficacy and steroid sparing effect [13–15].

**Figure 1:**
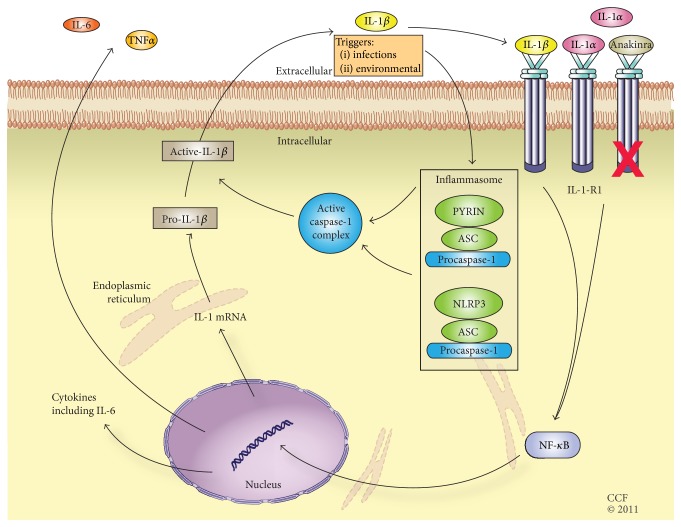
Mechanism of action of anakinra. Both IL-1*α* and IL-1*β* act through IL-1 receptor 1 to stimulate the production of inflammatory cytokines and TNF*α* that lead to the inflammatory cascade. The inflammasome is a complex of distinct proteins which together convert inactive prointerleukin-1*β* to active IL-1*β*. Environmental and infectious triggers can mediate the formation of the inflammasome. Anakinra blocks IL-1 receptor 1, antagonizing the effects of both IL-1*α* and IL-1*β*. ASC: Apoptosis associated speck-like protein containing caspase activation and recruitment domain, IL: interleukin, IL-1-R1: interleukin-1 receptor 1, NF-*κ*B: nuclear factor kappa-light-chain-enhancer of activated B cells, NLRP3: NOD-Like Receptor containing pyrin domain 3.

Anakinra, a short acting interleukin-1 (IL-1) receptor antagonist has been reported to be of benefit in refractory IRP and has become a focus of much interest in the treatment of IRP [21] (Figure 1). Although IL-1 has been known to play a pivotal role in inflammation for more than a decade, its clinical importance in numerous disease states has only more recently been elucidated [22]. The two distinct IL-1 genes,* IL1A* and* IL1B,* encode IL-1*α* and IL-1*β*, respectively. IL-1*α* and IL-1*β* bind to the universally expressed cell surface receptor, IL-1 receptor type-1, triggering a cascade of inflammatory mediators [23]. The precursor form of IL-1*α* is expressed in keratinocytes, mucous membrane epithelial cells, and organs such as the liver and vascular endothelium of healthy individuals. During pathological states, IL-1*α* moves to the cell surface or is released after cell death to activate IL-1 receptors in adjacent cells, which begins the cascade of sterile inflammation. IL-1*β*, on the other hand, is not expressed in healthy individuals, but it requires a stimulus such as microbial products or other chemokines to trigger its transcription in monocytes, tissue macrophages, and dendritic cells via the inflammasome [24]. IL-1 drives the inflammatory cascade in classic autoinflammatory conditions such as TRAPS and FMF and also plays a significant role in systemic onset juvenile idiopathic arthritis and in autoimmune diseases, such as rheumatoid arthritis [25]. Furthermore, children born with a loss-of-function mutation of the naturally occurring endogenous IL-1 receptor antagonist (IL-1Ra) succumb to early death due to widespread sterile inflammation caused by unopposed IL-1*β* function [26]. By antagonizing the action of IL-1 receptor, anakinra blocks the action of IL-1*α* and IL-1*β* and thus prevents the cascade of sterile inflammation in pathological state and in the assembly of the inflammasome.

Anakinra is used to treat rheumatoid arthritis and received United States Food and Drug Administration (FDA) approval for this condition in 2001 [27]. Anakinra is also FDA approved for use in autoinflammatory disease neonatal-onset multisystem inflammatory disease (NOMID), a severe subtype of cryopyrin-associated periodic syndrome (CAPS) [28]. Anakinra has been used off label in multiple autoimmune and autoinflammatory diseases including systemic onset juvenile idiopathic arthritis, colchicine-resistant FMF, TRAPS, and gout with trials underway for various conditions underlying chronic inflammatory states such as cardiovascular disease and diabetes [28–35].

Picco et al. first demonstrated the efficacy of anakinra in 3 pediatric patients with IRP in 2009 [21] (Table 1). All patients had rapid reversal of symptoms, with normalization of inflammatory markers on initiation of anakinra and were able to rapidly taper and discontinue steroids with continued use. Pericarditis promptly recurred when anakinra was discontinued, further supporting its efficacy. Picco et al. postulated that an unidentified autoinflammatory state underlies IRP in a subset of patients, who might respond to anakinra. The patients were treated with a dose of 1–1.25 mg/kg/day of anakinra and remained in remission while on therapy for a follow-up of 3-4 months.

Isolated reports followed in pediatric patients with IRP demonstrating similar results [16, 18]. In a recent small multicenter study, Finetti et al. studied 12 children and 3 adults (median age: 18 years, range: 8–60 years) with colchicine-resistant and steroid-dependent IRP treated with anakinra and confirmed an impressive 95% reduction in IRP flares over a median follow-up of 39 months (range: 6–57 months) [17]. All patients had dramatic clinical improvement within an average 2-day time period and were weaned off steroids at a median duration of 2 months (range: 0–7 months). At a median follow-up of 39 months, all 15 patients were in remission with 10 on anakinra monotherapy and 5 off all medications. Anakinra was used at a dose of 1-2 mg/kg/day in this study. Other than minor skin reactions, this group had no serious adverse events.

Two short case series from an investigational group in Greece, consisting of 3 and 10 adult patients (the series with 10 patients included follow-up data from the initial series with 3 patients) first demonstrated similar results in adult patients with steroid-dependent IRP [19, 36]. The reported adult dose was 100–150 mg/day by subcutaneous injection, with a regimen of daily dosing for 6 months followed by alternate dosing in the next 6 months for 7/10 patients. Paralleling the pediatric studies, adult patients had rapid clinical response, which allowed tapering of steroids, and had 70% recurrence rate on discontinuation of anakinra. Transient elevation of transaminases was noted in a minority of the patients (1 of 3 and 1 of 10 in each case series), along with minor skin reactions without any other serious adverse reactions reported. More recently, Jain et al. from the Mayo clinic in United States shared their experience with anakinra in the management of refractory pericarditis among 13 adults (12 IRP and 1 patient with postinfarction pericarditis) [37]. Anakinra at a dose of 100 mg once daily subcutaneously was used due to debilitating symptoms in spite of prednisone or as a steroid sparing agent. Rapid clinical improvement within 2–5 days occurred that was complete in most of the patients, with 1 patient having partial improvement. Patients were followed for a median duration of about 23 months, at the end of which 2 patients continued to require low dose prednisone, while the remaining were weaned off all other medications. Two patients were weaned off anakinra, while the remaining continued to require anakinra either at the initial dose or as a reduced dose (50 mg/day or 50 mg every other day). A recent systematic review concluded that anakinra was highly effective without significant side effects in patients with IRP with the major drawback being recurrences on discontinuation [38].

Preliminary data from the first comparative study was presented recently by Brucato et al. in 2015 [20]. Their research team reported on a double blind placebo controlled withdrawal trial. They enrolled a total of 21 patients with IRP (mean number of recurrences: 6.6) who were currently on corticosteroids. All the enrolled patients were treated initially with anakinra for 2 months, following which 11 patients were randomly assigned to continue anakinra for additional 6 months or until a pericarditis flare and 10 patients were assigned to placebo. All 10 patients assigned to placebo had a recurrence, but remarkably none of the patients assigned to the anakinra for 6 months had a recurrence. Minor side-effect of skin site reaction was noted in the majority of the patient, while 1 patient developed herpes zoster while on treatment and another developed ischemic optic neuropathy of unclear etiology. Although we await complete report of this study and other randomized controlled trials, these results are promising for the management of this otherwise difficult to treat population.

## 4. Conclusion

Although these preliminary reports appear promising, certain caveats remain. First, these are small case series and trials, and further larger randomized controlled trials are required to establish a definite efficacy for anakinra. Second, DMARDs such as methotrexate, the role of which has yet to be studied in IRP, could be an important addition to the standardized treatment of IRP [39]. Third, a systematic study found that anakinra was associated with a higher risk of serious infection compared with control treatments [27]. In a recent French study, anakinra was associated with serious adverse reactions in 9% of patients (children and adults), predominantly secondary to infections [40]. Along with the commonly reported minor local skin reactions, isolated cases of anaphylaxis and an interstitial granulomatous reaction have also been reported [41]. Fourth, the role of the longer acting canakinumab, a selective, fully human, anti-interleukin-1*β* monoclonal antibody, needs to be elucidated for increased efficacy and decreased adverse reactions [42]. Lastly, in some patients with recurrent pericarditis who respond to anakinra, the role of genetic studies in establishing the diagnosis of autoinflammatory syndromes needs to be explored. In the quest to find the ideal steroid sparing therapy, the etiopathogenesis of the recurrences in IRP require further clarification of the complex interaction between environmental triggers and genetic susceptibility.

## Highlights

The take-home points are as follows.

1. The etiopathogenesis of idiopathic recurrent pericarditis is unclear; evidence points to an immune mechanism.
2. The immune mechanism underlying idiopathic recurrent pericarditis shares characteristics of both autoimmune and autoinflammatory diseases.
3. Preliminary studies show promising results with anakinra in the management of a subset of patients with idiopathic recurrent pericarditis who are refractory to conventional management.
4. Larger and long-term studies are needed to address the safety and efficacy of anakinra in recurrent pericarditis.

## Figures and Tables

**Table 1:** Studies on the efficacy of anakinra in idiopathic recurrent pericarditis.

Study	Number of patients	Age (years)	Anakinra dose	Follow-up	Adverse reaction	Note
Picco et al., 2009 [21]	3	12, 13, 14	1–1.25 mg/kg/day	6 months	None reported	Pericarditis recurred in all 3 patients when anakinra was stopped but resolved on reinitiation of anakinra

Vassilopoulos et al., 2012 [19]	3	26, 36, 19	100–150 mg	6–15 months	Transient transaminase elevation in 1 patient	(i) Anakinra discontinued without recurrence in 1 patient after 6 weeks (ii) One recurrence after discontinuation of anakinra, which was treated with colchicine and ibuprofen (iii) One recurrence after anakinra discontinuation treated with anakinra reinstitution

Lazaros et al., 2014 [36]	10	Median: 39 (24–60 years)	100 mg daily for 6 months followed by 100 mg on alternate days for 6 months	8–40 months	Minor skin reactions (6/10)	(i) Date from 3 patients from [16] were included (ii) In the remaining 7 patients, 5/7 had recurrence after stopping anakinra. Anakinra was reinitiated in 4 and recurrence was treated conservatively in 1

Finetti et al., 2014 [17]	15	Median: 16 (8–60 years)	1-2 mg/kg/day	39 months (6–57 months)	Minor skin reactions	At follow-up, 10 were on anakinra monotherapy and 3 were weaned off all medications

Jain et al., 2015 [37]	13	Median: 49 (33–73 years)	100 mg daily	16.8 months (1.3–24 months)	Transient local reaction in 4 patients	(i) Anakinra weaned without recurrence in 2 patients (ii) Eleven patients remained on anakinra at follow-up

Brucato et al., 2015, RCT [20]	21	Mean: 45.4 years	100 mg daily (adult); 2 mg/kg/day (child)	2 months, all patients; 6 months, RCT	Transient local reaction (20) Herpes zoster (1) Optic neuropathy (1)	(i) None of the patients on the anakinra group had recurrences (ii) All patients on placebo developed recurrent pericarditis

RCT: randomized controlled trial.

## References

[1] Imazio M. (2014). Treatment of recurrent pericarditis. *Revista Espanola de Cardiologia*.

[2] Imazio M., Demichelis B., Parrini I. (2005). Management, risk factors, and outcomes in recurrent pericarditis. *American Journal of Cardiology*.

[3] Cantarini L., Lopalco G., Selmi C. (2015). Autoimmunity and autoinflammation as the yin and yang of idiopathic recurrent acute pericarditis. *Autoimmunity Reviews*.

[4] Doria A., Zen M., Bettio S. (2012). Autoinflammation and autoimmunity: bridging the divide. *Autoimmunity Reviews*.

[5] Caforio A. L. P., Brucato A., Doria A. (2010). Anti-heart and anti-intercalated disk autoantibodies: evidence for autoimmunity in idiopathic recurrent acute pericarditis. *Heart*.

[6] Imazio M., Brucato A., Doria A. (2009). Antinuclear antibodies in recurrent idiopathic pericarditis: prevalence and clinical significance. *International Journal of Cardiology*.

[7] Lazaros G., Karavidas A., Spyropoulou M. (2011). The role of the immunogenetic background in the development and recurrence of acute idiopathic pericarditis. *Cardiology*.

[8] Rigante D., Cantarini L., Imazio M. (2011). Autoinflammatory diseases and cardiovascular manifestations. *Annals of Medicine*.

[9] Cantarini L., Rigante D., Merlini G. (2014). The expanding spectrum of low-penetrance TNFRSF1A gene variants in adults presenting with recurrent inflammatory attacks: clinical manifestations and long-term follow-up. *Seminars in Arthritis and Rheumatism*.

[10] Pankuweit S., Wädlich A., Meyer E., Portig I., Hufnagel G., Maisch B. (2000). Cytokine activation in pericardial fluids in different forms of pericarditis. *Herz*.

[11] Fowler N. O. (1990). Recurrent pericarditis. *Cardiology Clinics*.

[12] Rodriguez de la Serna A., Guindo Soldevila J., Marti Claramunt V., Bayes de Luna A. (1987). Colchicine for recurrent pericarditis. *The Lancet*.

[13] Imazio M., Belli R., Brucato A. (2014). Efficacy and safety of colchicine for treatment of multiple recurrences of pericarditis (CORP-2): a multicentre, double-blind, placebo-controlled, randomised trial. *The Lancet*.

[14] Vianello F., Cinetto F., Cavraro M. (2011). Azathioprine in isolated recurrent pericarditis: a single centre experience. *International Journal of Cardiology*.

[15] Moretti M., Buiatti A., Merlo M. (2013). Usefulness of high-dose intravenous human immunoglobulins treatment for refractory recurrent pericarditis. *American Journal of Cardiology*.

[16] Camacho-Lovillo M., Méndez-Santos A. (2013). Successful treatment of idiopathic recurrent pericarditis with interleukin-1 receptor antagonist (Anakinra). *Pediatric Cardiology*.

[17] Finetti M., Insalaco A., Cantarini L. (2014). Long-term efficacy of interleukin-1 receptor antagonist (anakinra) in corticosteroid-dependent and colchicine-resistant recurrent pericarditis. *Journal of Pediatrics*.

[18] Scardapane A., Brucato A., Chiarelli F., Breda L. (2013). Efficacy of an interleukin-1*β* receptor antagonist (Anakinra) in idiopathic recurrent pericarditis. *Pediatric Cardiology*.

[19] Vassilopoulos D., Lazaros G., Tsioufis C., Vasileiou P., Stefanadis C., Pectasides D. (2012). Successful treatment of adult patients with idiopathic recurrent pericarditis with an interleukin-1 receptor antagonist (anakinra). *International Journal of Cardiology*.

[20] Brucato A., Imazio M., Maestroni S. (2015). Anakinra in patients with cortico-dependent idiopathic recurrent pericarditis: a randomised double-blind placebo-controlled withdrawal trial. *Arthritis & Rheumatology*.

[21] Picco P., Brisca G., Traverso F., Loy A., Gattorno M., Martini A. (2009). Successful treatment of idiopathic recurrent pericarditis in children with interleukin-1*β* receptor antagonist (anakinra): an unrecognized autoinflammatory disease?. *Arthritis & Rheumatism*.

[22] Dinarello C. A., Wolff S. M. (1993). The role of interleukin-1 in disease. *The New England Journal of Medicine*.

[23] Dinarello C. A., Simon A., van der Meer J. W. M. (2012). Treating inflammation by blocking interleukin-1 in a broad spectrum of diseases. *Nature Reviews Drug Discovery*.

[24] Zeft A. S., Spalding S. J. (2012). Autoinflammatory syndromes: fever is not always a sign of infection. *Cleveland Clinic Journal of Medicine*.

[25] Dinarello C. A. (2011). Interleukin-1 in the pathogenesis and treatment of inflammatory diseases. *Blood*.

[26] Aksentijevich I., Masters S. L., Ferguson P. J. (2009). An autoinflammatory disease with deficiency of the interleukin-1-receptor antagonist. *The New England Journal of Medicine*.

[27] Bresnihan B., Alvaro-Gracia J. M., Cobby M. (1998). Treatment of rheumatoid arthritis with recombinant human interleukin-1 receptor antagonist. *Arthritis and Rheumatism*.

[28] Koné-Paut I., Galeotti C. (2014). Anakinra for cryopyrin-associated periodic syndrome. *Expert Review of Clinical Immunology*.

[29] Zeft A., Hollister R., Lafleur B. (2009). Anakinra for systemic juvenile arthritis: the rocky mountain experience. *Journal of Clinical Rheumatology*.

[30] Larsen C. M., Faulenbach M., Vaag A. (2007). Interleukin-1-receptor antagonist in type 2 diabetes mellitus. *The New England Journal of Medicine*.

[31] Van Tassell B. W., Toldo S., Mezzaroma E., Abbate A. (2013). Targeting interleukin-1 in heart disease. *Circulation*.

[32] Calligaris L., Marchetti F., Tommasini A., Ventura A. (2008). The efficacy of anakinra in an adolescent with colchicine-resistant familial Mediterranean fever. *European Journal of Pediatrics*.

[33] So A., De Smedt T., Revaz S., Tschopp J. (2007). A pilot study of IL-1 inhibition by anakinra in acute gout. *Arthritis Research and Therapy*.

[34] Simon A., Bodar E. J., van der Hilst J. C. H. (2004). Beneficial response to interleukin 1 receptor antagonist in traps. *American Journal of Medicine*.

[35] Vitale A., Rigante D., Lucherini O. M. (2013). Biological treatments: new weapons in the management of monogenic autoinflammatory disorders. *Mediators of Inflammation*.

[36] Lazaros G., Vasileiou P., Koutsianas C. (2014). Anakinra for the management of resistant idiopathic recurrent pericarditis. Initial experience in 10 adult cases. *Annals of the Rheumatic Diseases*.

[37] Jain S., Thongprayoon C., Espinosa R. E. (2015). Effectiveness and safety of anakinra for management of refractory pericarditis. *The American Journal of Cardiology*.

[38] Lazaros G., Imazio M., Brucato A. Anakinra: an emerging option for refractory idiopathic recurrent pericarditis: a systematic review of published evidence. *Journal of Cardiovascular Medicine*.

[39] Brucato A., Brambilla G., Adler Y., Spodick D. H. (2005). Recurrent pericarditis: therapy of refractory cases. *European Heart Journal*.

[40] Rossi-Semerano L., Fautrel B., Wendling D. (2015). Tolerance and efficacy of off-label anti-interleukin-1 treatments in France: a nationwide survey. *Orphanet Journal of Rare Diseases*.

[41] Baldo B. A. (2014). Side effects of cytokines approved for therapy. *Drug Safety*.

[42] Ruperto N., Brunner H. I., Quartier P. (2012). Two randomized trials of canakinumab in systemic juvenile idiopathic arthritis. *The New England Journal of Medicine*.

